# Feasibility of superimposed supine cycling and lower body negative pressure as an effective means of prolonging exercise tolerance in individuals experiencing persisting post‐concussive symptoms: Preliminary results

**DOI:** 10.1113/EP091677

**Published:** 2024-08-05

**Authors:** Raelyn Javra, Joel S. Burma, Nathan E. Johnson, Jonathan D. Smirl

**Affiliations:** ^1^ Cerebrovascular Concussion Lab, Faculty of Kinesiology University of Calgary Calgary Alberta Canada; ^2^ Sport Injury Prevention Research Centre, Faculty of Kinesiology University of Calgary Calgary Alberta Canada; ^3^ Human Performance Laboratory, Faculty of Kinesiology University of Calgary Calgary Alberta Canada; ^4^ Libin Cardiovascular Institute of Alberta University of Calgary Calgary Alberta Canada; ^5^ Alberta Children's Hospital Research Institute University of Calgary Calgary Alberta Canada; ^6^ Hotchkiss Brain Institute University of Calgary Calgary Alberta Canada; ^7^ Integrated Concussion Research Program University of Calgary Calgary Alberta Canada

**Keywords:** cerebral blood flow, concussion treatment, exercise, lower body negative pressure, mild traumatic brain injury, persisting post‐concussion symptoms, supine cycling

## Abstract

To examine the feasibility, utility and safety of superimposed lower body negative pressure (LBNP) and tilt during supine cycling in individuals suffering from persisting post‐concussive symptoms (PPCS). Eleven individuals aged 17–31 (6 females/5 males) participated in two randomized separate visits, 1 week apart. A ramp‐incremental test was performed during both visits until volitional failure. Visits included no pressure (control) or LBNP at −40 Torr (experimental) with head‐up tilt at 15 degrees (females) or 30 degrees (males). Transcranial Doppler ultrasound was utilized to quantify middle cerebral artery velocity (MCAv), while symptom reports were filled out before and 0, 10, and 60 min post‐exertion. Ratings of exertion and overall condition followed similar trends for participants across both tests. The relative increase in MCAv was blunted during the experimental condition (8%) compared to control (24%), while a greater heart rate (17 beats/min) was achieved during the LBNP condition (*P =* 0.047). Symptom severity at the 0 and 10 min post‐exertion time points displayed negligible‐to‐small effect sizes between conditions (Wilcoxon's *r *< 0.11). Symptom reporting was lower at the 60 min post‐exertion time point with these displaying a moderate effect size (Wilcoxon's *r* = 0.31). The combination of LBNP and tilt during supine cycling did not change the participants’ subjective interpretation of the exertional test but attenuated the hyperpnia‐induced vasodilatory MCAv response, while also enabling participants to achieve a higher heart rate during exercise and reduced symptoms 1 h later. As this protocol is safe and feasible, further research is warranted in this area for developing PPCS treatment options.

## Abstract (highlights)

- **What is the central question of this study?**
  What are the feasibility, safety and utility of combining head‐up tilt with lower body negative pressure during supine cycling for blunting the increase in cerebral blood velocity seen during moderate‐intensity exercise in individuals experiencing persisting post‐concussion symptoms?
- **What is the main finding and its importance?**
  Although no differences were found in symptoms between conditions within the first 10 min following exertion, symptom severity scores showed a clinically meaningful reduction 60 min following the experimental condition compared to the non‐experimental control condition.

## 1 INTRODUCTION

The American Academy of Neurology (AAN) defines concussion as a form of mild traumatic brain injury, resulting in alterations to mental status that may or may not involve a loss of consciousness (Giza et al., 2013). Although not explicitly stated by the AAN, the Concussion in Sport Group (CISG) sixth consensus statement refers to the effects of concussions as being transient, whereas those of mild traumatic brain injuries (mTBIs) are seen to be semipermanent or permanent (Patricios et al., 2023). It is estimated 3.8 million concussions occur in the USA per year and approximately 1% of Canadians (∼500,000) sustain a concussion every year (Harmon et al., 2013; Langer et al., 2020).

Most patients spontaneously recover from a concussion within 7–10 days; however, ∼30% of people (∼1.25 million annually in North America) report having persistent symptoms longer than 1 month post‐concussion, known as persisting post‐concussion symptoms (PPCS) (Yeates et al., 2023). As such, PPCS poses a significant burden on the individual, their supporting environment as well as the health care economic system (Fu et al., 2016). For example, Fu et al. (2016) noted the estimated lifetime costs from traumatic brain injuries in Ontario in 2009 alone was approximately $945 million. Therefore, developing novel treatment strategies is imperative to help ensure these individuals can return to as normal life as possible, as soon as possible.

Overwhelming evidence in recent years has supported that moderate amounts of prescribed exercise in addition to physical activity expedited recovery following concussion (Chizuk et al., 2021; Leddy et al., 2019, 2021, 2023; Miutz et al., 2022). Furthermore, recent research has shown to support the benefit of active rehabilitation programmes for those suffering from persistent symptoms after mTBI (Howell et al., 2022; Kurowski et al., 2017; Mercier et al., 2021; Vidal et al., 2012). While individuals may experience temporary symptom exacerbation, chronic beneficial exercise‐based adaptations for individuals suffering from concussion include decrease in resting sympathetic activation, increased parasympathetic tone, lowered inflammation, increased production of brain‐derived neurotropic factor, improved systemic blood pressure regulation, reduced free‐radicals, reduced reactive oxidative species, and elevated cerebral blood flow (CBF) (Lucas et al., 2015; Rutschmann et al., 2021). Studies following concussion where exercise has been prescribed to target submaximal intensity up to approximately 60% of maximal oxygen uptake correspond to a ∼20%–30% increase in CBF due to hyperpnoea induced‐vasodilatation (Ogoh & Ainslie, 2009; Smith & Ainslie, 2017). However, despite this influx of research supporting exercise for concussion recovery, oftentimes those suffering from PPCS face barriers when it comes to exercise, as the action of performing exercise often triggers the onset of symptom exacerbation, such as headaches, dizziness and general discomfort.

According to the Monro–Kellie doctrine, the brain is enclosed in a confined space of the parenchyma, the cerebrospinal fluid and intracerebral blood, where an increase in CBF during exercise could apply additional pressure within the enclosed skull (Monro, 1783; Wilson, 2016). The known exacerbation of physiologically based concussion symptoms such as headaches, dizziness or pressure in the head, as a result of exercise, may be in part due to elevated CBF and intracranial pressure associated with the Monro–Kellie doctrine (Tan et al., 2014). Nevertheless, previous work has noted this increase during exercise can be blunted with the superimposed effect of lower body negative pressure (LBNP) and head‐up tilt in healthy adults (Burma, Seok et al., 2023; Goswami et al., 2018; Yoshimoto et al., 1994). Furthermore as concussion is known to cause dysfunction of the autonomic and cerebrovascular system (Leddy et al., 2007; Pertab et al., 2018), it is unknown if the combinatory effects of LBNP and head‐up tilt have a similar effect for individuals with PPCS. For example, if the cerebrovascular response to blood pressure is attenuated (i.e., cerebral autoregulation), a smaller degree of LBNP may be required compared to healthy controls with unaltered cerebral autoregulation (Kostoglou et al., 2016; Smirl et al., 2022; Wright, Smirl, Bryk, Fraser et al., 2018; Wright, Smirl, Bryk, & van Donkelaar, 2018). It is well known that exercise is important for overall health (Booth et al., 2012); there is an overwhelming amount of research demonstrating that lifelong exercise increases life span, delays chronic illness, improves cognition and mental health, and improves overall quality of life (Ruegsegger & Booth, 2018). Consequently, beyond the direct benefits of concussion recovery, it is important for individuals to be able to exercise for a variety of reasons. Due to the aforementioned barriers to exercise, those who suffer from ongoing concussion symptoms may take years to return to exercise, if they ever do, and do not reap the overall benefits of exercise.

As mentioned, prior research has proven the utility of LBNP and head‐up tilt to decrease cerebral blood flow in healthy adults (Burma, Seok et al., 2023; Goswami et al., 2018; Yoshimoto et al., 1994); however, further research is required to see if this method will allow those suffering from concussion symptoms to reap the benefits of exercise for recovery. Prior to doing so, it is imperative to ensure utility and safety of implementing these for this vulnerable population. Prior work from this research group has shown it is feasible to employ LBNP to blunt the typical increases in cerebral blood velocity (CBv) in young healthy adults (Miutz et al., 2023) and there were minimal‐to‐no alterations in CBv when applying this protocol across the menstrual cycle (Johnson et al., 2023). This protocol development was expanded to include the effects of head‐up tilt to enable CBv to return to baseline levels during cycling with a reduced level of negative pressure (−40 mmHg) and set up the foundation for the current protocol (−40 mmHg LBNP combined with a 15 degree tilt for females and a 30 degree tilt for males) (Burma, Seok et al., 2023).

Therefore, the purpose of the current investigation was to explore the feasibility, safety, and utility of combining head‐up tilt with LBNP during supine cycling to understand if this blunts the increase in CBF seen during moderate‐intensity exercise for individuals suffering from ongoing concussion symptoms at least a month post‐injury. It was hypothesized that (1) individuals suffering from ongoing concussion‐like symptoms would safely be able to complete superimposed with the combined use of LBNP and head‐up tilt, and (2) individuals would self‐report with a lower symptom burden following the experimental LBNP and head‐up tilt condition compared to the control condition.

## 2 METHODS

### 2.1 Ethical approval

The current study received ethical approval from the Calgary's Conjoint Health Research Ethics Board (REB21‐1517). Before study commencement, all protocols were thoroughly explained, instrumentation was explained, all questions were answered, and written consent was obtained prior to data collection. All study protocols, except for a priori database registration, were conducted in accordance with the Declaration of Helsinki (2013 revised).

### 2.2 Participants and study design

Participants consisted of 11 individuals diagnosed with PPCS (6 females, 5 males) aged 17–31 (23.5 ± 4.3 years), who had a physician‐diagnosed concussion and who were experiencing clinical symptoms associated with concussion that have extended beyond the expected recovery window for concussion (day 14 post‐injury) (Patricios et al., 2023). The range between injury to testing varied from 17 to 2219 days (∼6 years), with a median of 310 days. Mechanism of injury for the individuals consisted of sport‐related concussion (*n* = 6), a fall (*n* = 2), or a motor vehicle collision (*n* = 2). No participants disclosed the use of any prescribed medications. To minimize the likelihood of external confounding factors influencing the outcome metrics, a randomized crossover design was used. This augments the internal validity for a given study design, as participants act as their own control (Mills et al., 2009), thereby limiting any potential influences from injury mechanism or time since injury as the same individuals performed both arms of the investigation in a randomized order. Before all testing sessions, participants were asked to refrain from exercise, smoking, alcohol, vaping and caffeine for 12 h prior to maintain consistency between testing days and ensure results were accurate (Burma, Copeland, Macaulay, Khatra, Wright et al., 2020; Burma, Copeland, Macaulay, Khatra, Bouliane et al., 2020; Kennedy et al., 2022). A prior investigation using a similar study design measuring CBv (an index for CBF) (Skow et al., 2022) found high reliability across the menstrual cycle (Johnson et al., 2023). In addition, other studies assessing the influence of hormonal changes on cerebral autoregulation demonstrated minimal variation, and therefore testing was completed based upon convenience (Favre & Serrador, 2019; Johnson et al., 2023; Korad et al., 2022).

### 2.3 Instrumentation

**FIGURE 1:**
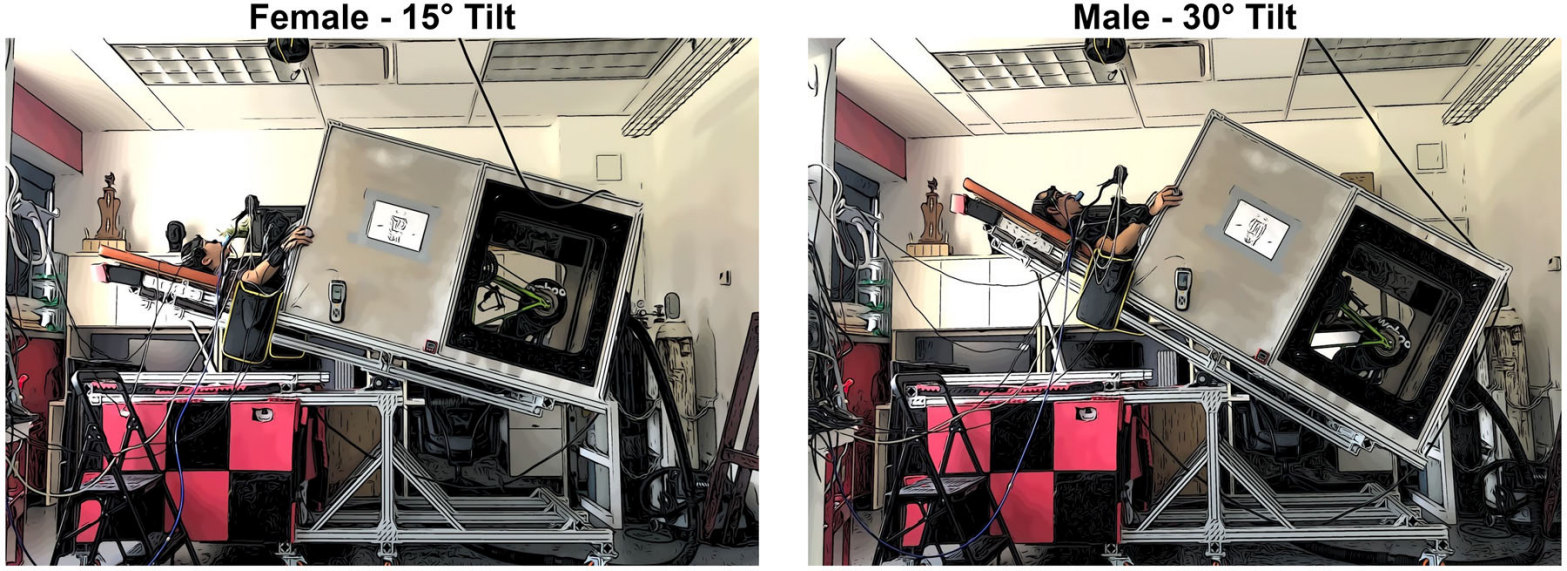
A representative photo displaying the lower body negative pressure chamber and the tilt used for female (15 degrees) and male (30 degrees) participants.

The LBNP chamber was custom‐built with a supine cycle ergometer situated inside containing a bicycle seat with length‐adjustable pedals. Participants were fitted into the opening of the chamber with a wooden plank around their waist and secured with a plastic sleeve and band to ensure a comprehensive pressure seal. An illustration of the LBNP chamber can be seen in Figure 1. During both tests, bilateral middle cerebral artery velocities (MCAv), heart rate (HR), blood pressure (BP), and the partial pressure end‐tidal value of carbon dioxide ($P_{\mathrm{ETC}O_{2}}$) were monitored and collected. MCAv was measured using transcranial Doppler ultrasound (TCD) with 2 MHz probes placed and secured in the temporal region using a fitted headframe (DWL USA, Inc, San Juan Capistrano, CA, USA). Once vessels were identified and confirmed (Purkayastha & Sorond, 2012; Willie et al., 2014), the velocity and depth of the waveforms were recorded. These were used as a reference on the second visit to minimize sonographer‐induced between‐day variation. Heart rate was monitored using a Polar heart rate monitor (Polar Electro Oy, Kempele, Finland), fitted using a chest strap, as well as a three‐lead electrocardiogram (FE 231 BioAmp; ADInstruments, Colorado Springs, CO, USA). Beat to beat BP was monitored using a Finapres (Finometer NOVA; Finapres Medical Systems, Amsterdam, The Netherlands) and was corrected at heart level (Omboni et al., 1993; Sammons et al., 2007). Capnography was used to measure oxygen and carbon dioxide values on a breath‐by‐breath basis (ML206; ADInstruments). All data were time synced, collected at 1000 Hz, and stored for offline analyses using LabChart (LabChart Pro Version 8, ADInstruments).

### 2.4 Experimental protocol

Recruited individuals were invited to participate in two testing sessions occurring at the same time of day, mitigating influences of diurnal variation (Burma, Copeland, Macaulay, Khatra, & Smirl, 2020). Testing sessions were randomized using a coin‐flip and separated by 7 days. This ensured the first randomized test would have minimal impact on the second. Before experimentation on day 1, participants completed the PAR‐Q+ (Warburton et al., 2019), ensuring they were cleared to engage in exercise. Individuals then completed a baseline symptom evaluation via the Sport Concussion Assessment Tool 5/6 (Echemendia et al., 2017, Echemendia, Brett, Broglio, Davis, Giza, Guskiewicz, Harmon, Herring, Howell, Master, McCrea et al., 2023; Echemendia, Brett, Broglio, Davis, Giza, Guskiewicz, Harmon, Herring, Howell, Master, Valovich McLeod et al., 2023), rating of perceived exertion (RPE) via the Borg scale (Borg, 1982) and overall condition via an 11 point visual analogue scale (Leddy et al., 2018). Participants’ age, height, body mass and mechanism of injury were recorded. Once equipment was attached, participants were tilted to 30 degrees for males and 15 degrees for females, as per Burma, Seok et al. (2023). For a detailed explanation on the development of the LBNP protocol employed in the current investigation, the reader is directed to Miutz et al. (2023), Johnson et al. (2023) and Burma, Seok et al. (2023). In brief, Miutz et al. (2023) demonstrated it is feasible to employ LBNP to blunt the typical increases in CBv in young healthy adults. Johnson et al. (2023) built upon this work by examining the CBv response to the LBNP recommendations from the prior study across the menstrual cycle and showed there were minimal‐to‐no effects from four testing sessions performed across the menstrual cycle. Finally, Burma, Seok et al. (2023) expanded the prior work to include the effects of head‐up tilt to enable CBv to return‐to‐baseline levels during cycling with a reduced level of negative pressure (−40 mmHg) and set up the foundation for the current protocol (−40 mmHg LBNP combined with a 15 degree tilt for females and a 30 degree tilt for males). After instrumentation was completed in the supine position, tilt was applied, and a minimum of 2 min of buffer time was given to allow for the hemodynamic fluid shift associated with orthostatic challenges to reach steady‐state (Smith et al., 1994). Three minutes of resting baseline measures were then obtained for all physiological variables, while participants quietly rested. The protocol began at the individual's calculated power (in watts), which was incrementally increased during each minute stage based on body mass consistent with previous supine cycling tests (Miutz et al., 2023):

Females:WattStage=0.15×kgofbodymass

Males:WattStage=0.20×kgofbodymass

At the end of each minute, participants reported their RPE and their overall condition. Throughout the entirety of the test, volunteers cycled at a cadence between 60 and 80 revolutions per minute. Participants continued cycling until (1) volitional fatigue was achieved or (2) their overall condition increased 3 points on the visual analogue scale (Leddy et al., 2018). The two testing sessions were identical, except 1 day was completed during −40 Torr of LBNP (experimental condition) while the other had no LBNP applied (control condition). To minimize the influence of the vacuum associated sounds with the experimental condition, the sides of the LBNP chamber remained open (did not allow a negative pressure to be generated), while the vacuum was turned on at the same setting. This minimized a difference in noise between testing conditions, and the potential for participants to report ‘sensitivity to noise’ during the LBNP compared to the control condition. Upon the completion of the test, participants completed the SCAT symptom evaluation immediately, 10 min and 60 min following exertion. Total symptom scores (TSS [/22]) and symptom severity scores (SSS [/132]) were calculated from the Symptom Evaluation component of a SCAT5 assessment for each time the participant completed the symptom evaluation (Echemendia et al., 2017). Each of the 22 individual symptoms is rated on a 0–6 Likert graded scale.

### 2.5 Data processing

For both tests, the MCAv was chosen to represent CBv as it is responsible for delivery of ∼70% of blood to the brain (including delivery to the motor cortex) (Zarrinkoob et al., 2015). When both MCAv were well maintained during the test sessions, the average of the two were employed in the analysis (80%); however, if there was a decrease in signal quality for one of the vessels, the stronger of the two were employed for analysis. During the exercise test, the last 20 s were taken from each stage and averaged to determine the MCAv.

CBv and BP values were calculated across the cardiac cycle (Burma, Copeland, Macaulay, Khatra, & Smirl, 2020; Burma, Copeland, Macaulay, Khatra, Wright et al., 2020; Burma, Rattana et al., 2023). Systolic and diastolic values were obtained as the maximum and minimum value from each pulsatile waveform, respectively. Mean values were calculated as the average of all data points calculated across each waveform. The relative percentage increase in physiological measures during each condition was calculated as the difference from baseline to the greatest value during exercise, with an example as:

$$\mathrm{Relative}\;\mathrm{MCAv}\;\mathrm{increase}\;=\frac{\left(\mathrm{Maximal}\;\mathrm{MCAv}-\mathrm{Baseline}\;\mathrm{MCAv}\right)}{\mathrm{Baseline}\;\mathrm{MCAv}}\;\;\times \;100$$

**FIGURE 2:**
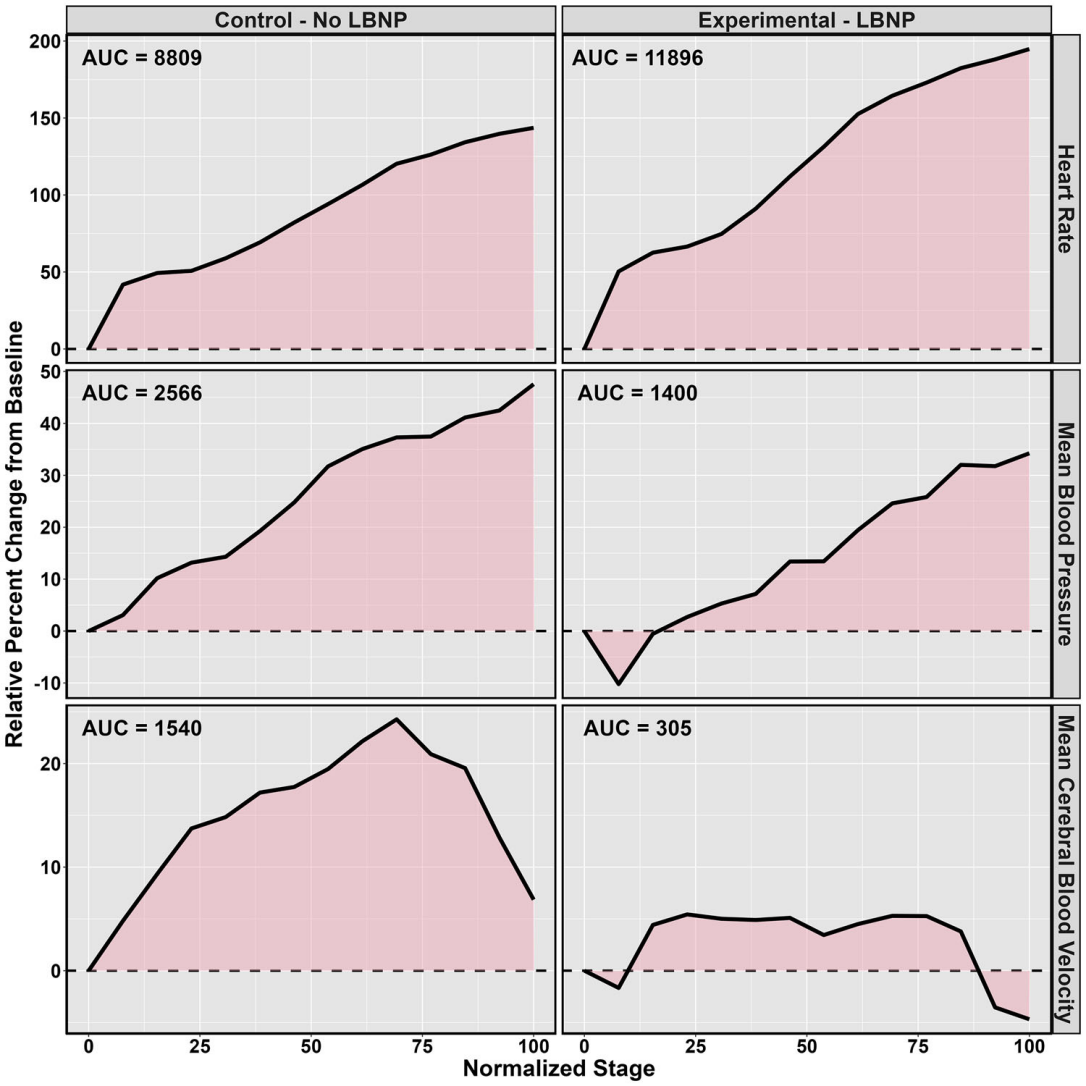
The area under the curve (AUC) produced across the control no lower body negative pressure (LBNP) and the experimental LBNP conditions for heart rate, mean blood pressure and mean cerebral blood velocity from one participant. The AUC is expressed as the accumulation of each physiological signal across the exercise tests in beats per minute (heart rate), millimetres of mercury (mean blood pressure) and centimetres per second (mean cerebral blood velocity).

Further, to control for the differing number of stages completed between tests, each stage was normalized to the final stage. For example, if an individual completed 15 stages of the cycle test, stage 5 would be normalized to 33% of the test duration. This allowed for area under the curve (AUC) calculations to be computed for all physiological variables using the normalized stage and physiological values (Figure 2). In brief, AUC describes the region bounded by a physiological increase or decrease during the exercise bout relative to baseline, that is, bound between two *y*‐axes (i.e., baseline and final stage).

For the SCAT5 TSS and SSS scores, delta scores were calculated for the three post‐exertion tests (i.e., immediate, 10 and 60 min) by subtracting this from the baseline value. Thus, a positive delta would mean symptoms are higher following the exertion test compared to baseline, while a negative indicated symptoms were lower at the follow‐up time points.

### 2.6 Statistical analyses

R Studio (Version 2022.7.1.554) was used to perform all statistical analyses. To calculate the between‐day variability for all physiological measures, coefficient of variation (CoV) metrics were computed. Wilcoxon's signed rank test with effect sizes and 95% confidence intervals was performed for the absolute and relative final physiological values obtained during the last stage of each exercise test, the maximum relative increase calculation (diastolic, mean and systolic CBv and diastolic, mean and systolic BP), the AUC calculations, and symptom scores (i.e., TSS and SSS). Effect size thresholds for these were based upon: <0.10 (negligible), 0.10–0.30 (small), 0.30–0.50 (moderate) and >0.50 (large) (Lakens, 2013). Due to growing concern within the physiological and biomedical literature, inferences were computed from a combination of both *P‐*values and effect sizes (Halsey, 2019; Sullivan & Feinn, 2012; Thiese et al., 2016). Using *P*‐values and effect sizes in combination provides a complete statistical analysis by not only indicating whether a result is due to chance but also quantifying the magnitude of the observed effect, providing clinically relevant comparisons/interpretations (Halsey, 2019; Sullivan & Feinn, 2012; Thiese et al., 2016). Finally, in concussed individuals, it is somewhat expected that those who are able to achieve a higher heart rate on a maximal test may report a greater degree of symptomology (Paniccia et al., 2018). To better understand this, the AUCs for heart rate were plotted against SSS, with correlation values derived via Spearman's rank correlation coefficients (ρ) (Schober et al., 2018). This enabled an understanding of whether higher heart rate values were associated with SSS, as it is expected the experimental condition will produce a slightly different physiological response due to the external application of the LBNP stimulus. α was set a priori at 0.05. Data are presented as boxplots (median and interquartile range (IQR)).

## 3 RESULTS

Of the 11 individuals recruited, one participant dropped out following the first visit as they withdrew from the study (9%). Drop‐out occurred because of acute symptom exacerbation and the participant did not feel comfortable to complete the subsequent session. No difference was found in the total duration between each test (LBNP: median = 14.5 min, IQR = 13–16 min; control: median = 13.5 min, IQR = 12.5–15.5 min; *P* = 0.732). No differences were found for RPE and overall condition between conditions (*P *> 0.622, Wilcoxon's *r* < 0.12; negligible‐to‐small).

Good‐to‐excellent levels of between‐day CoVs were found for CBv measures (<5%), physiological measures (<7%) and clinical symptoms (<10%), noting any differences between tests were not explained due to baseline variation between exercise tests. At the end of the final stage, greater values were seen for heart rate (*P* = 0.047, Wilcoxon's *r* = 0.44; 95% CI: 0.08–0.71) during LBNP compared to control, while blood pressure was lower (*P* = 0.043, Wilcoxon's *r* = 0.46; 95% CI: 0.13–0.75). Mean MCAv similarly had a moderate effect size (Wilcoxon's *r* = 0.37; 95% CI: 0.02–0.69), albeit this was not significant (*P* = 0.105). No other physiological measure was different at the end of both exertional tests (*P *> 0.218, Wilcoxon's *r* < 0.28; negligible‐to‐small). Table 1 demonstrates the mean increase above baseline for all physiological measures and the corresponding effect sizes. Of interest, blood pressure, heart rate and diastolic MCAv displayed moderate effect sizes, while mean MCAv had a large effect size (Table 1). A larger AUC was found for heart rate (*P* = 0.023, Wilcoxon's *r* = 0.51; 95% CI: 0.10–0.79) during the LBNP condition, while a smaller diastolic MCAv (*P* = 0.034, Wilcoxon's *r* = 0.48; 95% CI: 0.07–0.74) and mean MCAv (*P *< 0.001, Wilcoxon's *r* = 0.79; 95% CI: 0.61–0.85) were present. All other variables were not significant and displayed a negligible‐to‐small effect size (*P *> 0.190, Wilcoxon's *r* < 0.27) aside from mean blood pressure, which did have a moderate effect size (Wilcoxon's *r* = 0.31; 95% CI: 0.04–0.69).

**TABLE 1:** Differences in physiological responses during cycling with either no lower body negative pressure (LBNP; control) or with concurrent application of −40 Torr LBNP in 10 individuals (5 females and 5 males).

	Control—no LBNP	Experimental—LBNP	Comparisons and effect size
Absolute final value			
Diastolic MCAv (cm/s)	53 ± 16	46 ± 12	*P* = 0.218, *r *= 0.29 (95% CI: 0.04–0.73)
Mean MCAv (cm/s)	85 ± 20	72 ± 16	*P* = 0.105, *r *= 0.37 (95% CI: 0.02–0.69)
Systolic MCAv (cm/s)	126 ± 24	113 ± 25	*P* = 0.315, *r *= 0.24 (95% CI: 0.01–0.68)
Diastolic BP (mmHg)	78 ± 8	77 ± 9	*P* = 0.853, *r *= 0.05 (95% CI: 0.00–0.51)
Mean BP (mmHg)	120 ± 12	109 ± 8	***P* = 0.043, *r *= 0.46 (95% CI: 0.13–0.75)**
Systolic BP (mmHg)	202 ± 31	192 ± 27	*P* = 0.436, *r *= 0.19 (95% CI: 0.01–0.54)
Heart rate (bpm)	168 ± 21	185 ± 12	***P* = 0.047, *r *= 0.44 (95% CI: 0.08–0.71)**
$P_{\mathrm{ETC}O_{2}}$ (mmHg)	35 ± 8	32 ± 8	*P* = 0.631, *r *= 0.12 (95% CI: 0.01–0.51)
Relative final value (%)			
Diastolic MCAv	100 ± 21	88 ± 14	*P* = 0.063, *r *= 0.42 (95% CI: 0.03–0.80)
Mean MCAv	116 ± 15	97 ± 9	***P* = 0.004, *r *= 0.63 (95% CI: 0.25–0.85)**
Systolic MCAv	117 ± 18	108 ± 11	*P* = 0.280, *r *= 0.22 (95% CI: 0.01–0.66)
Diastolic BP	124 ± 16	117 ± 13	*P* = 0.481, *r *= 0.25 (95% CI: 0.01–0.64)
Mean BP	145 ± 14	130 ± 10	***P* = 0.015, *r *= 0.54 (95% CI: 0.14–0.80)**
Systolic BP	156 ± 19	149 ± 22	*P* = 0.089, *r *= 0.39 (95% CI: 0.01–0.66)
Heart rate	230 ± 48	272 ± 45	*P* = 0.063, *r *= 0.42 (95% CI: 0.07–0.70)
$P_{\mathrm{ETC}O_{2}}$	86 ± 19	82 ± 19	*P* = 0.739, *r *= 0.09 (95% CI: 0.00–0.58)
Largest relative increase (%)			
Diastolic MCAv	118 ± 15	107 ± 8	*P* = 0.063, *r *= 0.42 (95% CI: 0.08–0.72)
Mean MCAv	124 ± 11	108 ± 5	***P* < 0.001, *r *= 0.74 (95% CI: 0.46–0.85)**
Systolic MCAv	128 ± 15	122 ± 10	*P* = 0.280, *r *= 0.25 (95% CI: 0.02–0.72)
Diastolic BP	126 ± 16	121 ± 12	*P* = 0.481, *r *= 0.17 (95% CI: 0.01–0.56)
Mean BP	145 ± 14	132 ± 10	***P* = 0.035, *r *= 0.47 (95% CI: 0.07–0.80)**
Systolic BP	158 ± 19	159 ± 20	*P* = 0.529, *r *= 0.15 (95% CI: 0.01–0.65)
Heart rate	230 ± 48	272 ± 45	*P* = 0.063, *r *= 0.42 (95% CI: 0.05–0.76)
$P_{\mathrm{ETC}O_{2}}$	109 ± 11	110 ± 9	*P* = 0.631, *r *= 0.12 (95% CI: 0.01–0.60)
Cumulative area under the curve across the exercise test			
Diastolic MCAv (cm/s/test)	563 ± 950	‐328 ± 748	***P* = 0.034, *r *= 0.48 (95% CI: 0.07–0.74)**
Mean MCAv (cm/s/test)	1473 ± 778	111 ± 371	***P* < 0.001, *r *= 0.79 (95% CI: 0.62–0.85)**
Systolic MCAv (cm/s/test)	1624 ± 825	1183 ± 724	*P* = 0.247, *r *= 0.27 (95% CI:0.01–0.64)
Diastolic BP (mmHg/test)	945 ± 893	526 ± 609	*P* = 0.393, *r *= 0.20 (95% CI: 0.01–0.66)
Mean BP (mmHg/test)	1793 ± 694	1361 ± 687	*P* = 0.190, *r *= 0.30 (95% CI: 0.04–0.69)
Systolic BP (mmHg/test)	3136 ± 1137	3110 ± 1342	*P* = 0.739, *r *= 0.08 (95% CI: 0.00–0.58)
Heart rate (bpm/test)	6976 ± 2622	9710 ± 2565	***P* = 0.023, *r *= 0.51 (95% CI: 0.10–0.79)**
$P_{\mathrm{ETC}O_{2}}$ (mmHg/test)	103 ± 860	153 ± 852	*P* > 0.999, *r *= 0.01 (95% CI: 0.00–0.58)

*Note*: This was completed during tilt at 15 degrees for females and 30 degrees for males. Data are displayed as means ± standard deviation. Bolded comparisons signify a difference between the Control and Experimental Conditions. Abbreviations: BP, blood pressure; BPM, beats per minute; MCAv, middle cerebral artery velocity; $P_{\mathrm{ETC}O_{2}}$, partial pressure end‐tidal carbon dioxide.

**FIGURE 3:**
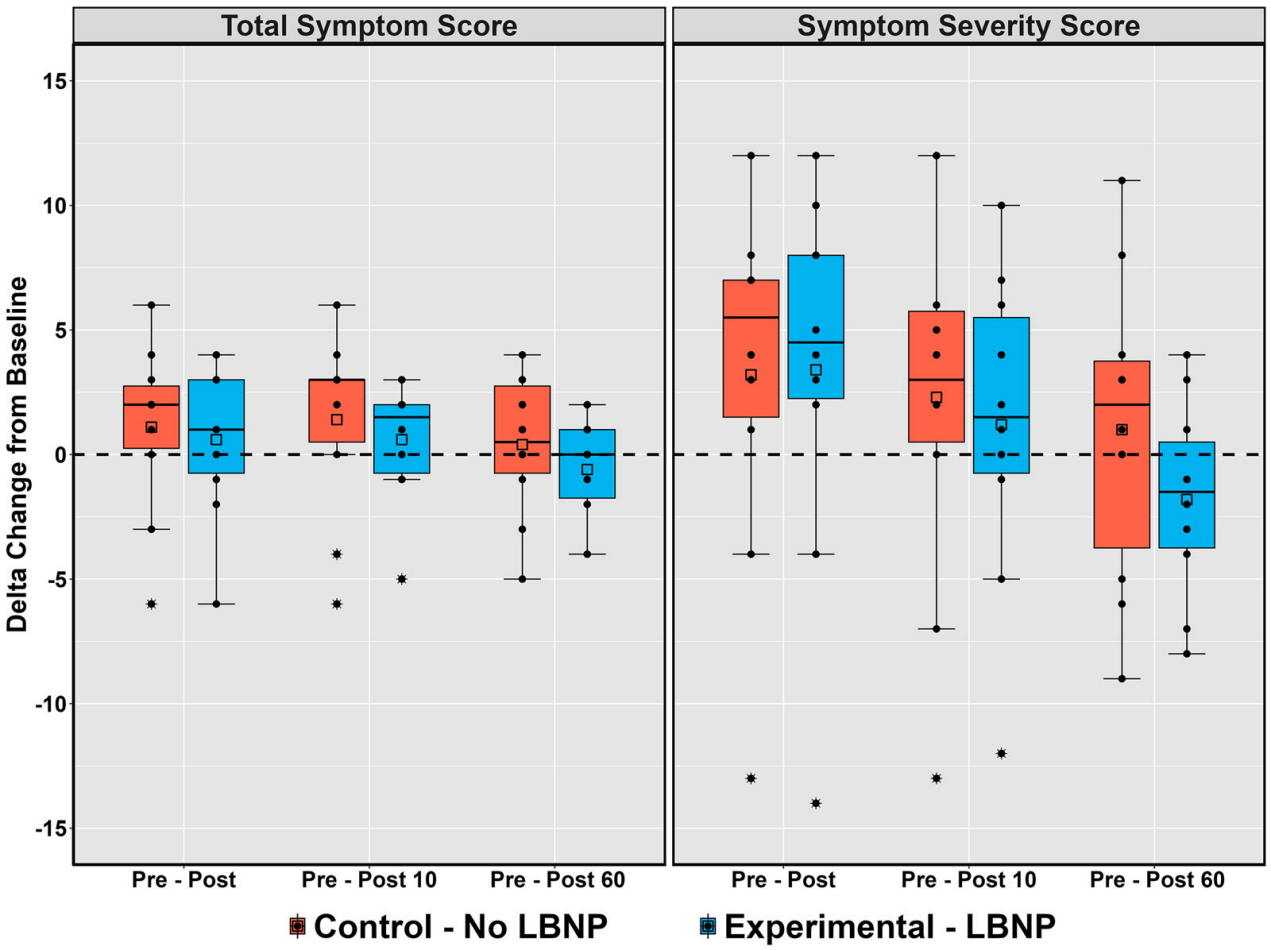
Boxplots with error bars detailing the change in total symptom scores and symptom severity scores obtained with the symptom evaluation portion of the Sport Concussion Assessment Tool 5 between baseline and post‐exercise. Symptoms were captured at baseline, immediately, 10 min and 60 min following test termination in 10 individuals (5 female and 5 male). LBNP, lower body negative pressure.

**FIGURE 4:**
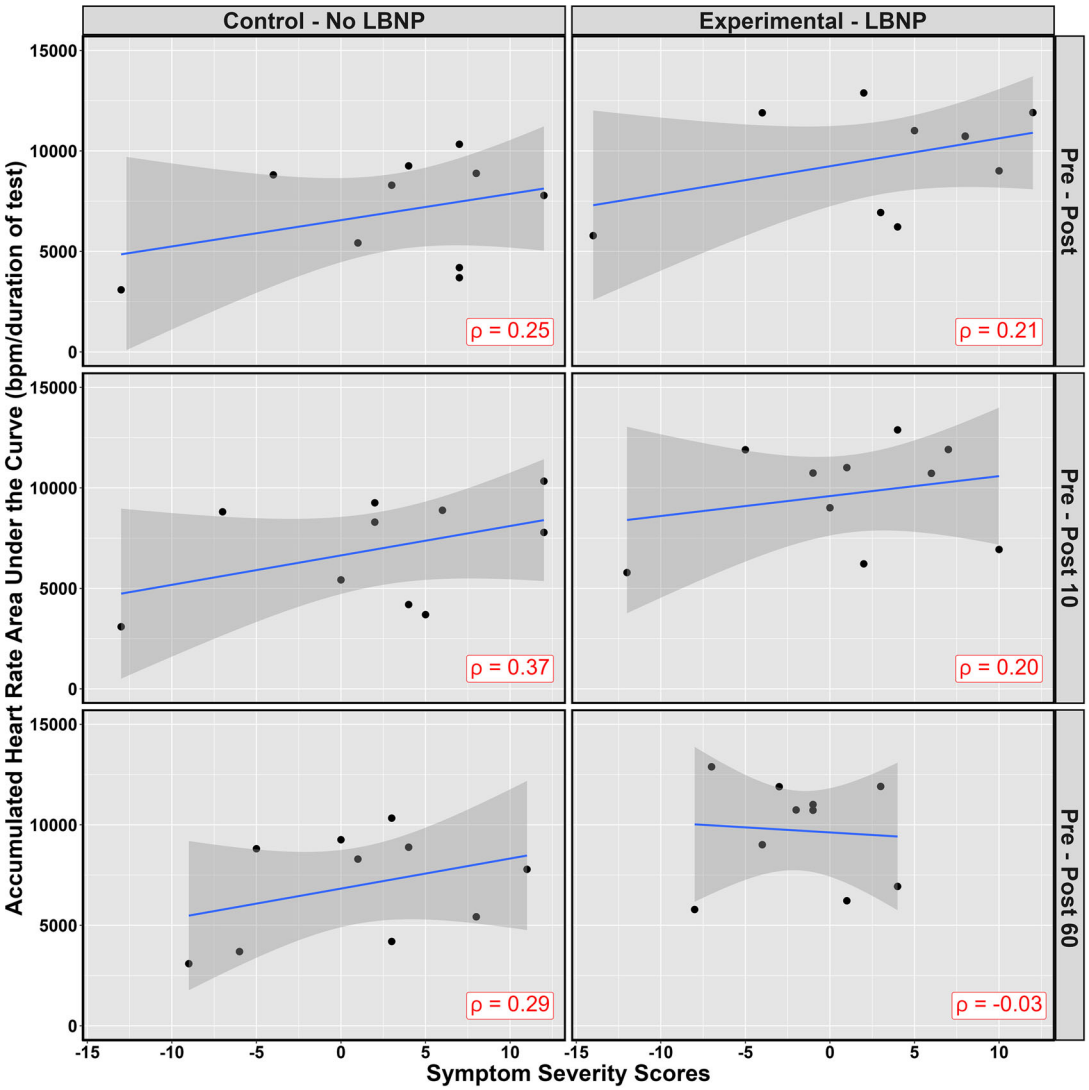
The accumulated area under the curve for heart rate as a function of symptom severity scores. Symptoms were captured at baseline, immediately, 10 min and 60 min following test termination in 10 individuals (5 female and 5 male), where the post‐exercise time points were compared to baseline levels. In general, a positive trend is found, where increasing levels of heart rate are correlated with a greater symptom severity, aside from the Pre–Post 60 comparison.

Both TSS (Wilcoxon's *r* = 0.09; 95% CI: 0.00–0.54) and SSS (Wilcoxon's *r* = 0.05; 95% CI: 0.01–0.54) had a negligible effect size between conditions immediately following the exertion tests (Figure 3). At the post 10 min time point, TSS (Wilcoxon's *r* = 0.29; 95% CI: 0.03–0.69) and SSS (Wilcoxon's *r* = 0.11; 95% CI: 0.01–0.56) had a small effect size (Figure 3). The TSS (Wilcoxon's *r* = 0.21; 95% CI: 0.01–0.62) and SSS (Wilcoxon's *r* = 0.31; 95% CI: 0.01–0.63) had small and moderate effect sizes at the 60 min post‐exertion time point, respectively, with these values being lower in the LBNP condition (experimental) compared to the control condition (Figure 3). Interestingly, Figure 4 shows the correlation of SSS with the accumulated heart AUC during the tests. Positive ρ correlations were found across all post‐exercise time points for the control condition and only the immediate and 10 min post‐exertion time points for the LBNP condition (Figure 4). At the 60 min post‐exertion time point following LBNP, an absence of a ρ correlation was found between symptoms and heart rate accumulated during the exertional test (Figure 4). Table 2 shows the means of each individual symptom severity score as well as the mean total severity score at the three separate time points between the control and experimental groups. Of interest, pressure in the head, fatigue and total symptom scores had notable results (Table 2). At the 60 min time point, pressure in the head, fatigue, and total symptom score different significantly from pre‐testing in the experimental group (+0.14, −0.31 and −2.43, respectively) compared to the control group (+0.72, +0.57 and +0.57, respectively).

**TABLE 2:** Differences in average symptoms pre, post, post‐10 and post‐ 60 min with either no lower body negative pressure (LBNP; control) or with concurrent application of −40 Torr LBNP in 10 individuals (5 females and 5 males).

	Control—No LBNP				Experimental—LBNP			
Symptom	PRE	POST‐0	POST‐10	POST‐60	PRE	POST‐0	POST‐10	POST‐60
Headache	1.71	2.14	2.00	2.14	1.57	2.14	2.00	1.86
Pressure in the head	1.14	2.29	2.29	1.86	1.00	2.00	2.00	1.14
Neck pain	1.86	1.29	1.71	1.14	1.00	0.71	0.57	1.29
Nausea/vomiting	0.00	0.14	0.14	0.14	0.00	0.57	0.14	0.00
Dizziness	0.00	0.43	0.86	0.14	0.14	1.29	0.71	0.29
Blurred vision	0.14	0.29	0.29	0.14	0.14	0.57	0.29	0.14
Balance problems	0.00	0.00	0.29	0.00	0.00	0.14	0.00	0.14
Sensitivity to light	0.43	0.57	0.29	0.57	0.57	0.14	0.14	0.43
Sensitivity to noise	0.43	0.29	0.14	0.14	0.29	0.00	0.00	0.29
Feeling slowed down	0.57	0.57	1.00	0.57	0.43	0.57	1.00	0.29
Feeling like ‘In a Fog’	0.71	0.57	0.57	0.71	0.57	0.43	1.14	0.29
‘Don't Feel Right’	0.57	0.57	0.57	0.57	0.57	0.43	0.71	0.43
Difficulty concentrating	0.86	0.86	0.86	1.00	1.14	1.43	1.14	0.57
Difficulty remembering	0.86	0.57	0.57	0.71	1.14	0.86	0.71	0.43
Fatigue/low energy	0.57	2.29	1.14	1.14	1.00	2.00	1.14	0.29
Confusion	0.00	0.00	0.14	0.00	0.14	0.00	0.00	0.00
Drowsiness	0.43	0.57	0.14	0.14	0.43	0.29	0.14	0.29
Trouble falling asleep	0.86	0.43	0.43	0.43	1.00	0.71	0.71	0.43
More emotional than usual	0.14	0.14	0.14	0.14	0.14	0.14	0.14	0.14
Irritability	0.57	0.57	0.57	0.57	0.14	0.14	0.14	0.57
Sadness	0.29	0.29	0.29	0.29	0.14	0.14	0.14	0.00
Nervous/anxious	0.14	0.29	0.14	0.29	0.29	0.14	0.14	0.14
**TSS**	**7.43**	**8.57**	**9.29**	**8.29**	**7.71**	**8.29**	**8.43**	**7.00**
**SSS**	**12.29**	**15.14**	**14.57**	**12.86**	**11.86**	**14.86**	**13.14**	**9.43**

*Note*: This was completed during tilt at 15 degrees for females and 30 degrees for males. Bolded values are the summed Total Symptom Score (TSS) and Severity Symptom Score (SSS) from the above 22 symptoms.

## 4 DISCUSSION

The current study used a randomized crossover design to evaluate if the application of a provocative exercise test on individuals suffering from PPCS would lead to a short‐term increase in symptoms relative to standard progressive exercise tests commonly used in clinical practice for concussion management (Chizuk et al., 2022; Haider, Johnson et al., 2019; Haider, Leddy et al., 2019; Leddy et al., 2018, 2021). In general, RPE and overall condition were similar between conditions, demonstrating the addition of LBNP to a common exertion test post‐concussion did not change the subjective difficulty or make individuals feel worse, despite attaining a higher overall heart rate during the exercise bout. Additionally, the application of LBNP resulted in CBv peaking only 8% on average, compared to 24% during the control condition. Finally, while no differences were found in symptoms between conditions within the first 10 min following exertion, SSS showed a clinically meaningful reduction 60 min following the LBNP condition compared to the no‐LBNP control condition. Additionally, there was a smaller increase in pressure in the head at the 60 min time point compared to the pre‐test evaluation in the experimental group compared to the control. Fatigue and total symptom score were reduced significantly (−0.31 and −2.43, respectively) with the use of LBNP compared to an increase in both without it (+0.57 and +0.57, respectively). This demonstrates an overall reduction in symptoms at the 60 min time point, as well as the conclusion that participants felt like they subjectively had more energy after exercise with the use of LBNP and head‐up tilt. This is important as previous research has shown fatigue to be one of the key factors in PPCS (Norrie et al., 2010). Conclusively, this proof‐of‐concept study shows promising results regarding the employment of this combinatory mechanism on the feasibility and safety for individuals with PPCS; however, further work is warranted to expand these findings in concussed participants while considering age, sex, concussion history and other confounding factors.

### 4.1 Physiological underpinnings

Concussion has been described as a microstructural, metabolic and physiological injury to the brain that results in numerous signs and/or symptoms (Giza & Hovda, 2001, 2014). Common symptoms following a concussion include, but are not limited to, headaches, fatigue, pressure in the head, depression, difficulty concentrating, and so forth.  It has been proposed these symptoms are potentially linked to numerous aspects of the neurometabolic cascade, which is characterized as elevated glutamate release, influx of calcium into the neurons, a transient hypermetabolic glycolytic state, mitochondria dysfunction and the potential for neuronal apoptosis (Giza & Hovda, 2001, 2014). Additionally, concussion has been shown to impact the functioning of the cerebral endothelial lining within both the main conduit vessels and the microvasculature. These disturbances have been proposed to be linked to the short‐term increase in symptom‐reporting of those with a concussion experience acutely following exercise (Tan et al., 2014). For example, during moderate‐intensity exercise, CBv will increase ∼20%–30% above baseline values (Ogoh & Ainslie, 2009; Smith & Ainslie, 2017). As the brain is in a fixed and non‐expandable cranium, an increase in blood volume inside this space will result in elevated intracranial pressure (Monro, 1783; Wilson, 2016) and may underline some of the aforementioned symptoms such as headaches and/or pressure in the head.

A previous study by Miutz et al. (2023) demonstrated that the 20%–30% increase in CBv during cycling exercise can be attenuated with the application of LBNP at −60 Torr independently. Subsequent work by Burma, Seok et al. (2023) noted the combination of LBNP at −40 Torr with 15 degrees head‐up tilt for females and 30 degrees for males, returned CBv to pre‐exercise baseline values. These two orthostatic manipulations work to shift blood into the lower extremities resulting in greater pooling in the capacitance vessels, causing reduced venous return (Goswami et al., 2018). To compensate for the lowered venous return and subsequent stroke volume, heart rate will increase to ensure cardiac output remains relatively constant (Vincent, 2008). The elevated heart rate associated with the LBNP condition thus is likely attributable to the physiological adaptions expected during the exercise. Previous work by Leddy et al. (2018) has noted an individual's heart rate threshold in the acute phase of injury is predictive of their time to symptom resolution. This unique exercise modality may hold the greatest utility in individuals who have a lower exertion‐induced symptom threshold, where they would be able to exercise at a greater heart rate threshold, obtaining these exercise‐related benefits (Ahlskog et al., 2011; Barnes, 2015; Bliss et al., 2021; Mandolesi et al., 2018) while potentially minimizing symptom exacerbation. Finally, the decreased pressure applied to the lower extremities also results in more arterial blood being sent to the working muscles, meaning these are supplied with more glucose and oxygen, which may make moderate‐intensity subjectively easier (Figures 2 and 4).

It is important that individuals suffering from ongoing concussion‐like symptoms can find a way to exercise as there is extensive research to suggest aerobic exercise attenuates cognitive impairments (Ahlskog et al., 2011; Barnes, 2015; Bliss et al., 2021; Mandolesi et al., 2018). Exercise causes an induction of neuronal‐growth and repair‐promoting factors such as brain‐derived neurotrophic factor (BDNF) circulation, neuroplasticity, increased proliferation of neuronal stem cells, reduced degeneration and apoptosis around the damaged area, and improved cognitive performance (Griesbach et al., 2014; Itoh et al., 2011a, 2011b; Jacotte‐Simancas et al., 2015; Kim et al., 2010). This influx of BDNF aids with angiogenesis and neurogenesis, which can help promote regrowth of potentially compromised neuronal wiring (Griffin et al., 2011; Stroth et al., 2009).

Moreover, often overlooked in concussion recovery are the unique interplay of biopsychosocial factors, where concussion recovery has been shown to be influenced by one's perception of their recovery (Register‐Mihalik et al., 2020). Exercise additionally results in the release of endorphins, triggering positive feelings in the body, which mitigates feelings of depression, anxiety, irritability and so forth (Harber & Sutton, 1984). Hence, being able to exercise for a greater duration and potentially at a higher dose–response for heart‐rate during the novel proposed protocol may enable a greater release of endorphins and ultimately a quicker recovery (Leddy et al., 2023). Nevertheless, this study focused on the feasibility of redistributing blood flow and further research is warranted to confirm these propositions.

### 4.2 Implications for future research

While this proof‐of‐concept study highlights promising applications for this intervention strategy, further research is warranted with larger, more diverse samples, where age, sex, mechanisms of injury, history of concussion and other factors could be investigated. Moreover, the present study only included individuals experiencing broad strokes of PPCS, and the current approach might be more appropriate for those experiencing PPCS as a result of physiologically derived symptom challenges such as exercise‐induced headaches or pressure‐in‐the‐head. Furthermore, expanding these findings to those acutely concussed would unveil additional utility of the proposed methods. Additionally, future research should investigate the effects on symptomology if the trials were to last the same duration and/or be performed over multiple exercise bouts. While the subjective rating of exertion was relatively similar between tests, participants anecdotally stated they found the exercise protocol easier during the LBNP condition; however, the waist strap for sealing the chamber, combined with the suction, made it slightly harder to breathe explaining why overall RPE followed similar trends. Fine tuning of the methodology used to create the chamber seal above the iliac crest may mitigate the concerns surrounding breathing difficulties and thus would increase the ability to determine RPE differences. Finally, effect sizes were reported throughout, enabling researchers to use these findings to ensure subsequent studies are adequately powered.

### 4.3 Limitations

The largest limitation of the current investigation is the small, heterogeneous sample size used. However, these findings provide encouraging preliminary results that future investigation can build upon. TCD uses ultrasound waves to measure the velocity of red blood cells, which has great temporal resolution and has low sensitivity to movement artifacts, enabling it to be used during exercise (Purkayastha & Sorond, 2012). However, the limitation of the TCD is that it assumes the diameter of the vessel remains constant to provide a surrogate for CBF (Ainslie & Hoiland, 2014). During moderate‐intensity exercise, changes in blood pressure and predominantly circulating carbon dioxide levels will likely lead to slight alterations in diameter. Nonetheless, minimal respiratory and cardiovascular differences were noted between conditions, and this likely had a nominal influence on the outcome, with LBNP leading to reduced symptoms at 60 min post‐exercise. A final limitation is no physiologically derived measures of cardiorespiratory fitness were assessed in the current investigation (Drapeau et al., 2019; Labrecque et al., 2017, 2019). However, this would not be feasible to have participants with PPCS to complete a $\overset{\cdot }{V_{O_{2}\max}}$ test, and thus would only be discernible following complete physiological recovery. As individuals acted as their own controls for this study, any potential differences in cardiovascular fitness are minimized. In addition to this, while the duration between injury and testing varied largely between participants, each participant acted as their own control and the protocols were administered in a randomized order thereby minimizing any potential effects of time since injury. The physiological responses to sub‐acute and long‐term effects of concussion vastly differ (Giza & Hovda, 2001). Nevertheless, the study design of the current investigation was a randomized crossover design where each participant acted as their own control and controlled for any covariates that would impact the promising outcome measures (Mills et al., 2009).

### 4.4 Conclusion

This proof‐of‐concept study aimed to elucidate the feasibility and utility of superimposed LBNP and tilt during supine cycling for those suffering from PPCS. Following concussion, some individuals are known to experience exertional‐based symptom exacerbation, which may be associated with an increase in CBv and augmented intracranial pressure according to the Monro–Kellie doctrine. The current findings noted no differences in the duration, RPE or overall condition change between the exertional tests; however, participants reported a reduction in total symptom severity and reported clinically relevant lowered number of symptoms, notably pressure in the head and fatigue, for an extended period following the LBNP experimental condition. These current findings show great utility for those suffering from PPCS and may provide them with the positive benefits of exercise (e.g., BDNF, neural stem cell proliferation, neurogenesis, etc.) and accelerate recovery. It may also impact future concussion rehabilitation (for PPCS and individuals in the acute stage of recovery from concussion) as this may provide a novel technique for individuals who are limited in their exercise capacity due to symptom exacerbation associated with physical exertion to overcome this barrier. Nonetheless, a larger sample size is needed to confirm these propositions, as well as looking at the confounding effects of sex, age, concussion history and mechanism of injury.

## AUTHOR CONTRIBUTIONS

Raelyn Javra: coneptualization; methology; formal analysis; investigation; writing—original draft; writing—review and editing; visualization. Joel S Burma: coneptualization; methology; formal analysis; investigation; writing—original draft; writing—review and editing; visualization. Nathan E. Johnson: investigation, writing—review and editing; visualization. Jonathan D. Smirl: coneptualization; methology; writing—review and editing; funding acquisition; supervision. All authors have read and approved the final version of this manuscript and agree to be accountable for all aspects of the work in ensuring that questions related to the accuracy or integrity of any part of the work are appropriately investigated and resolved. All persons designated as authors qualify for authorship, and all those who qualify for authorship are listed.

## CONFLICT OF INTEREST

None declared.

## Data Availability

The materials and data that support the findings of this study are available from the corresponding author (J.S.B.) upon reasonable request.
